# C-type natriuretic peptide modulates quorum sensing molecule and toxin production in *Pseudomonas aeruginosa*

**DOI:** 10.1099/mic.0.046755-0

**Published:** 2011-07

**Authors:** Anne-Sophie Blier, Wilfried Veron, Alexis Bazire, Eloïse Gerault, Laure Taupin, Julien Vieillard, Karine Rehel, Alain Dufour, Franck Le Derf, Nicole Orange, Christian Hulen, Marc G. J. Feuilloley, Olivier Lesouhaitier

**Affiliations:** 1 Laboratory of Cold Microbiology – Signals and Micro-environment EA 4312, University of Rouen, 55 Rue Saint Germain, 27000 Evreux, France; 2 Laboratoire de Biotechnologie et Chimie Marines, Université de Bretagne-Sud B.P. 92116, 56321 Lorient Cedex, France; 3 SIMA, UMR 6014 COBRA, University of Rouen, 27000 Evreux, France

## Abstract

*Pseudomonas aeruginosa* coordinates its virulence expression and establishment in the host in response to modification of its environment. During the infectious process, bacteria are exposed to and can detect eukaryotic products including hormones. It has been shown that *P. aeruginosa* is sensitive to natriuretic peptides, a family of eukaryotic hormones, through a cyclic nucleotide-dependent sensor system that modulates its cytotoxicity. We observed that pre-treatment of *P. aeruginosa* PAO1 with C-type natriuretic peptide (CNP) increases the capacity of the bacteria to kill *Caenorhabditis elegans* through diffusive toxin production. In contrast, brain natriuretic peptide (BNP) did not affect the capacity of the bacteria to kill *C. elegans*. The bacterial production of hydrogen cyanide (HCN) was enhanced by both BNP and CNP whereas the production of phenazine pyocyanin was strongly inhibited by CNP. The amount of 2-heptyl-4-quinolone (HHQ), a precursor to 2-heptyl-3-hydroxyl-4-quinolone (*Pseudomonas* quinolone signal; PQS), decreased after CNP treatment. The quantity of 2-nonyl-4-quinolone (HNQ), another quinolone which is synthesized from HHQ, was also reduced after CNP treatment. Conversely, both BNP and CNP significantly enhanced bacterial production of acylhomoserine lactone (AHL) [e.g. 3-oxo-dodecanoyl-homoserine lactone (3OC12-HSL) and butanoylhomoserine lactone (C4-HSL)]. These results correlate with an induction of *lasI* transcription 1 h after bacterial exposure to BNP or CNP. Concurrently, pre-treatment of *P. aeruginosa* PAO1 with either BNP or CNP enhanced PAO1 exotoxin A production, via a higher *toxA* mRNA level. At the same time, CNP led to elevated amounts of *algC* mRNA, indicating that *algC* is involved in *C. elegans* killing. Finally, we observed that in PAO1, Vfr protein is essential to the pro-virulent effect of CNP whereas the regulator PtxR supports only a part of the CNP pro-virulent activity. Taken together, these data reinforce the hypothesis that during infection natriuretic peptides, particularly CNP, could enhance the virulence of PAO1. This activity is relayed by Vfr and PtxR activation, and a general diagram of the virulence activation cascade involving AHL, HCN and exotoxin A is proposed.

## INTRODUCTION

*Pseudomonas aeruginosa* is a ubiquitous Gram-negative opportunistic pathogen that can infect different hosts including mammals (Rahme *et al.*, 1995), insects (Jander *et al.*, 2000), nematodes (Tan & Ausubel, 2000) and plants (Rahme *et al.*, 2000). In humans, *P. aeruginosa* causes serious infections in immunocompromised hosts, is one of the major micro-organisms responsible for nosocomial diseases (Govan & Deretic, 1996) and is the predominant cause of morbidity and mortality in cystic fibrosis patients (Adams *et al.*, 1998; Govan & Deretic, 1996).

During infection, the stress status of the host promotes the release and circulation of eukaryotic signal molecules facilitating their contact with bacteria. Some reports suggest that *P. aeruginosa* PAO1 may actively sense eukaryotic signal molecules (Lesouhaitier *et al.*, 2009), such as gamma-interferon (Wu *et al.*, 2005) or dynorphin (Zaborina *et al.*, 2007) which trigger bacterial virulence factor production. Natriuretic peptides represent a family of eukaryotic hormones which comprises at least three major members: atrial natriuretic peptide (ANP), brain natriuretic peptide (BNP) and C-type natriuretic peptide (CNP). These peptides are principally produced by atrial (ANP and BNP) and endothelial (CNP) cells (de Bold *et al.*, 1996; Suga *et al.*, 1992). It has been observed that BNP concentration is increased in human blood during septic shock (Post *et al.*, 2008; Rudiger *et al.*, 2006) and that plasma N-terminal pro-brain natriuretic peptide (NT-proBNP) increases in a model of systemic exposure to *Escherichia coli* endotoxin (or lipopolysaccharide, LPS) in healthy volunteers (Vila *et al.*, 2008). Moreover, LPS potently stimulates CNP secretion by endothelial cells (Suga *et al.*, 1993). Thus, it appears that bacteria are exposed to natriuretic peptides in the plasma during the host infection process. Natriuretic peptides possess a structure similar to cationic antimicrobial peptides (Bulet *et al.*, 2004) and have been first proposed as antimicrobial peptides active against a large spectrum of micro-organisms (Krause *et al.*, 2001). However, we have recently demonstrated that, at a micromolar range, neither human BNP nor CNP act on *P. aeruginosa* survival but are able to enhance the global cytotoxicity of the bacterium (Veron *et al.*, 2007). The sensitivity of *P. aeruginosa* to natriuretic peptides appears to be relayed by the activation of a cyclase leading to an increase of intra-bacterial cAMP concentration and to a stimulation of the Vfr global regulator (Veron *et al.*, 2007). This implication of the Vfr protein suggests that numerous virulence factors or bacterial physiological parameters may be regulated by CNP in *P. aeruginosa*.

To make its infectious process successful, *P. aeruginosa* uses a large arsenal of both secreted and cell-associated virulence factors (Kipnis *et al.*, 2006; Lyczak *et al.*, 2000). In addition, it has been proposed that *P. aeruginosa* outer membrane vesicles, which contain numerous secreted factors and cell-membrane-associated virulence factors, may represent another weapon used by this bacterial pathogen (Bomberger *et al.*, 2009; Kuehn & Kesty, 2005; Mashburn & Whiteley, 2005). In *Pseudomonas*, the regulation of the expression of a wide variety of survival and virulence mechanisms is controlled in a cell-density-dependent manner through a process called quorum sensing (QS) (Juhas *et al.*, 2005; Rumbaugh *et al.*, 2000). Global regulation through QS systems controls population behaviours and plays fundamental roles in bacterial pathogenicity (Fuqua *et al.*, 2001; Smith & Iglewski, 2003; Winzer & Williams, 2001). *P. aeruginosa* QS is principally represented by the Las and Rhl systems. LasI and RhlI synthesize their cognate signal molecules 3-oxo-dodecanoyl-homoserine lactone (3OC12-HSL) and butanoylhomoserine lactone (C4-HSL), respectively. These two diffusible molecules induce gene expression by binding to their respective receptor proteins, LasR and RhlR, which are also transcriptional activators (Pearson *et al.*, 1995). A third signal has been found to participate in the *P. aeruginosa* QS network, the quinolone 2-heptyl-3-hydroxyl-4-quinolone, termed *Pseudomonas* quinolone signal (PQS) (Pesci *et al.*, 1999). PQS fits the functional criteria of a QS signal, such as cell-density-dependent accumulation (Lépine *et al.*, 2003) and recognition by bacterial cells in which it triggers a specific transcriptional response (Camilli & Bassler, 2006). PQS is synthesized by the mono-oxygenase PqsH which converts the 2-heptyl-4-quinolone (HHQ) into PQS (Gallagher *et al.*, 2002). Since it is released from and taken up by bacteria, HHQ is also considered to act as an intercellular message molecule (Déziel *et al.*, 2004). PQS signalling plays an important role in *P. aeruginosa* pathogenesis because it regulates diverse target genes including those coding for elastase, rhamnolipid, the PA-IL lectin and pyocyanin, as well as influencing biofilm development (Diggle *et al.*, 2006).

Among *P. aeruginosa* secreted virulence factors, exotoxin A (ETA) appears to be essential for the success of bacterial infection (Fogle *et al.*, 2002; Hamood *et al.*, 1996; Storey *et al.*, 1998). The blue phenazine pigment pyocyanin is also an important exotoxin secreted by *P. aeruginosa* (Diggle *et al.*, 2003). This molecule is directly toxic to both prokaryotic (Hassan & Fridovich, 1980) and eukaryotic (Denning *et al.*, 1998) cells and also metazoans such as *Caenorhabditis elegans* (Mahajan-Miklos *et al.*, 1999). Moreover, pyocyanin induces apoptosis in human neutrophils, favouring the persistence of *P. aeruginosa* in host tissues and promoting bacterial host defence evasion (Usher *et al.*, 2002). Hydrogen cyanide (HCN) is a diffusible gas produced by *P. aeruginosa* and also represents a formidable toxic factor against eukaryotic organisms including *C. elegans* (Gallagher & Manoil, 2001) and humans (Anderson *et al.*, 2010).

In the present study, we examined the impact of natriuretic peptides on the global virulence of PAO1 using the infectious model *C. elegans*. The effect of natriuretic peptides on *P. aeruginosa* production of both major virulence factors and communication signal molecules was determined. Finally, we identified key steps of the molecular mechanism involved in CNP action on *P. aeruginosa*.

## METHODS

### 

#### Bacterial cultures and tested molecules.

*P. aeruginosa* PAO1 was obtained from an international collection (Veron *et al.*, 2007). *P. aeruginosa* MPAO1 and mutants obtained by transposon insertion (Jacobs *et al.*, 2003) were from the University of Washington (Seattle, WA, USA) (Table 1). Both strains were grown in ordinary nutrient broth no. 2 medium (ONB) (AES) at 37 °C. A single colony of *P. aeruginosa* was used to inoculate 5 ml ONB. An overnight culture was diluted at 1 : 100 and the tested molecules [hBNP (Sigma-Aldrich) and CNP (Polypeptide)] were added 2 h later. The density of the bacterial suspension was determined by measuring optical density at 580 nm using a spectrophotometer (ThermoSpectronics). The bacterial density and the absence of contamination were controlled by plating. Synthetic C4-HSL was purchased from Fluka-Sigma-Aldrich and synthetic 3OC12-HSL from Sigma-Aldrich.

**Table 1.** Strains used in this study

Strain	Characteristic	Reference or source
***P. aeruginosa***		
PAO1	Wild-type	Veron *et al.* (2007)
MPAO1	Wild-type	Holloway *et al.* (1979)
PW6882	MPAO1 mutant *rhlR*-B10 : : IS*lacZ*/hah	University of Washington Genome Center
PW3597	MPAO1 mutant *lasR*-B10 : : IS*lacZ*/hah	University of Washington Genome Center
PW3601	MPAO1 mutant *lasI*-F07 : : IS*lacZ*/hah	University of Washington Genome Center
PW6880	MPAO1 mutant *rhlI*-D03 : : IS*phoA*/hah	University of Washington Genome Center
PW2181	MPAO1 mutant *vfr*-G11 : : IS*lacZ*/hah	University of Washington Genome Center
PW2283	MPAO1 mutant *toxR/regA*-E04 : : IS*lacZ*/hah	University of Washington Genome Center
PW4833	MPAO1 mutant *ptxR*-G01 : : IS*lacZ*/hah	University of Washington Genome Center
PW9967	MPAO1 mutant *algC*-D07 : : IS*phoA*/hah	University of Washington Genome Center
PW2810	MPAO1 mutant *phnB*-H01 : : IS*lacZ*/hah	University of Washington Genome Center
***E. coli***		
OP50		Stiernagle (1999)

#### *C. elegans* synchronization and virulence assays.

The *C. elegans* wild-type Bristol strain N2 was obtained from the *Caenorhabditis* Genetics Center (Minneapolis, USA). *C. elegans* was maintained under standard culturing conditions at 22 °C on nematode growth medium (NGM; all per litre, 3 g NaCl, 2.5 g peptone, 17 g agar, 5 mg cholesterol, 1 ml 1 M CaCl_2_, 1 ml 1 M MgSO_4_, 25 ml 1 M KH_2_PO_4_) agar plates with *E. coli* OP50 as a food source (Sulston & Hodgkin, 1988). Synchronous cultures of worms were generated after exposure of the adult worm population to a sodium hypochlorite/sodium hydroxide solution as described previously (Stiernagle, 1999). The resulting eggs were incubated at 22 °C on an *E. coli* OP50 lawn until the worms reached the L4 (48 h) life stage (confirmed by light microscopy).

Pathogen lawns used for *C. elegans* survival assays were prepared by spreading 50 µl *P. aeruginosa* strains (control and treated) on 35 mm peptoneglucose/sorbitol (PGS; 1 % Bacto-peptone, 1 % NaCl, 1 % glucose, 0.15 M sorbitol, 1.7 % Bacto-agar) conditioned Petri dishes for fast-killing evaluation or on 35 mm NGM conditioned Petri dishes supplemented with 0.05 mg 5-fluoro-2′-deoxyuridine ml^−1^ for slow killing determination. This nucleotide analogue blocks the development of the *C. elegans* next generation via the inhibition of DNA synthesis, thus preventing offspring from the experimental animals. The plates were incubated overnight at 37 °C and then placed at room temperature for 4 h. Between 15 and 20 L4 synchronized worms were harvested with M9 solution (per litre, 3 g KH_2_PO_4_, 6 g NaHPO_4_, 5 g NaCl, 1 ml 1 M MgSO_4_), placed on the 35 mm assay Petri dishes and incubated at 22 °C. Worm survival was scored at 1 h, 24 h and each subsequent day, using an Axiovert S100 optical microscope (Zeiss) equipped with a Nikon digital camera DXM 1200F (Nikon Instruments). The worms were considered dead when they remained static without grinder movements for 20 s. The results are expressed as the percentage of living worms and are the average of three independent assays.

#### Pyocyanin assay.

*P. aeruginosa* PAO1 was grown at 37 °C, in shaking conditions at 180 r.p.m. in ONB medium with either physiological water (control), BNP or CNP (1 µM). Following 24 h incubation, bacterial cells were centrifugated at 8000 ***g*** for 10 min, and 2 ml of supernatant was extracted using 2 ml chloroform and re-extracted with 1 ml 0.5 M HCl. The pyocyanin concentration was determined by measurement of OD_520_.

#### HSL and PQS extraction and quantification.

C4-HSL and 3OC12-HSL were extracted and quantified by liquid chromatography-mass spectrometry (LC-MS-MS) according to Morin *et al.* (2003). PQS, HHQ and 2-nonyl-4-quinolone (HNQ) were extracted as the HSLs (Morin *et al.*, 2003) and characterized as described by Déziel *et al.* (2004) and Bazire *et al.* (2005). After the bacterial cells were removed from the growth medium by centrifugation (2500 ***g***, 10 min), the supernatants were extracted twice with one vol. HPLC-grade dichloromethane. The dichloromethane extracts were dried over anhydrous magnesium sulfate, filtered and evaporated. The residue was dissolved in HPLC-grade acetonitrile and analysed using LC-MS-MS. The proportion of each PQS-related molecule was quantified from the corresponding *m*/*z* [M+H]^+^ chromatograms by integration of peak areas.

#### Elastase assay.

The elastolytic activity of *P. aeruginosa* culture supernatants was determined by using the elastin Congo red (ECR) assay (Pearson *et al.*, 1997). *P. aeruginosa* PAO1 was grown at 37 °C, in shaking conditions at 180 r.p.m. in ONB medium and exposed to either physiological water (control), BNP or CNP (1 µM). After 3 h incubation, bacterial cells were centrifuged at 2500 ***g*** for 10 min and the supernatants were filtered. Triplicate 50 µl samples of culture filtrates were added to tubes containing 20 mg ECR (Sigma-Aldrich) and 1 ml buffer (0.1 M Tris, pH 7.2, 1 mM CaCl_2_). The samples were incubated for 18 h at 37 °C with rotation; they were then placed on ice and 0.1 ml 0.12 M EDTA was added. Insoluble ECR was removed by centrifugation, and the OD_495_ of the supernatant was measured.

#### Azocasein assay.

Proteinase secretion was quantified by the azocasein assay as outlined by Kessler *et al.* (1982) with modifications. Azocasein (Sigma-Aldrich) was dissolved to 5 mg ml^−1^ in assay buffer containing 0.1 M Tris/HCl (pH 8). The *P. aeruginosa* PAO1 culture was incubated for 3 h with either physiological water (control), BNP or CNP, and media were removed and centrifuged to pellet the cells. The azocasein solution (250 µl) was mixed with 250 µl bacterial supernatants and incubated in a 37 °C water bath for 30 min. The reactions were stopped by adding 500 µl 10 % TCA, and the reaction mixtures were allowed to stand at –20 °C for 10 min. Samples were then centrifuged for 5 min at 8000 ***g***, and 500 µl of each supernatant was added to 500 µl 0.5 M NaOH. *A*_440_, corresponding to released azocasein, was determined with a spectrophotometer.

#### HCN assay.

HCN concentration in bacterial culture medium was determined by polarographic analysis. Voltametric measurements were made using a Metrohm (Herisau, Swiss) 757 VA Computrace. Analysis was carried out in a three-electrode configuration using an Ag/AgCl 3 mol KCl l^−1^ reference, a platinum wire as a counter electrode and a multi-mode mercury electrode as a working electrode. All experiments were performed at a room temperature. Bacterial cell cultures were centrifuged for 10 min at 8000 ***g*** and filtered at 0.22 µm. The filtered supernatants were diluted in a 0.2 mol borate l^−1^ electrolyte (pH 10.2). The solution was purged for 3 min with nitrogen to remove dissolved oxygen and then for 20 s more between each cyanide addition. A potential scan was carried out in a negative direction from −0.1 to −0.5 V with a sweep rate of 10 mV s^−1^. The pulse amplitude was 0.05 V with a pulse duration of 0.04 s. The peak height of cyanide was measured at −200 mV in a differential pulse mode and the cyanide concentration was determined by adding four successive amounts of potassium cyanide standard (10 mg l^−1^).

#### ETA quantification.

The assay was done using a protocol adapted from Coligan *et al.* (2001). Briefly, throughout the assay, the 96-well microplates (MaxiSorp F, Nunc) were washed with PBST (0.02 %, v/v, Tween 20 in PBS). Each well was coated with 100 µl diluted goat anti-ETA antibody (0.25 mg ml^−1^ in 100 mM Na_2_HCO_3_) (Sigma-Aldrich) overnight at 4 °C. The plates were washed and treated with 1 mg BSA ml^−1^ in PBST for 1 h at 37 °C. The plates were then washed twice and incubated with supernatant fractions (100 µl per well) for 1 h at 37 °C. Serial dilutions (512–2 pg µl^−1^) of purified ETA (Sigma-Aldrich) in PBST were used as standards. The plates were washed six times and incubated for 1 h at 37 °C with rabbit anti-ETA (100 µl per well) (Sigma-Aldrich) diluted in PBST. The plates were then washed six times and incubated for 1 h at 37 °C with goat anti-rabbit IgG conjugated to horseradish peroxidase (Sigma-Aldrich). The plates were washed again six times, and bound ETA was revealed by incubation at 37 °C for 30 min with 100 µl TMB (ImmunoPure TMB substrate, Sigma-Aldrich). The reaction was stopped by adding 100 µl 2 M H_2_SO_4_ per well. *A*_450_ was read using an ELISA plate reader (Bio-Rad 680 XR). The values were standardized by dividing the amount of ETA (pg ml^−1^) from each supernatant fraction by the OD_580_ of the culture from which that fraction was obtained.

#### mRNA assay by quantitative reverse transcription-PCR (qRT-PCR).

RNA quantification was performed as described previously (Bazire *et al.*, 2010) from bacteria grown for 3 h in liquid ONB medium. The primers used are given in Supplementary Table S1 (available with the online version of this paper). 16S rRNA was used as an endogenous control (Corbella & Puyet, 2003). PCRs were performed in triplicate and the standard deviations were lower than 0.15 C_T_. The relative quantifications were obtained as described previously (Bazire *et al.*, 2009), using the comparative C_T_ (2^−ΔΔCT^) method (Livak & Schmittgen, 2001).

#### Statistical analysis.

For the killing assay, nematode survival was calculated by using the Kaplan–Meier method, and survival differences were tested for significance by using the log–rank test (GraphPad Prism version 4.0, GraphPad Software). For other results, each value reported for the assays is the mean measurement for a minimum of three independent preparations. The non-parametric Mann–Whitney test was used to compare the means within the same set of experiments.

## RESULTS

### Effect of natriuretic peptides on PAO1 virulence towards *C. elegans*

Depending on the experimental conditions, *P. aeruginosa* kills *C. elegans* in different ways. In a low-salt medium such as NGM, *P. aeruginosa* kills the nematodes over a period of a few days (‘slow killing’) by an infection-like process in which the bacteria accumulate in the worm intestines (Tan *et al.*, 1999).

**Fig. 1.**
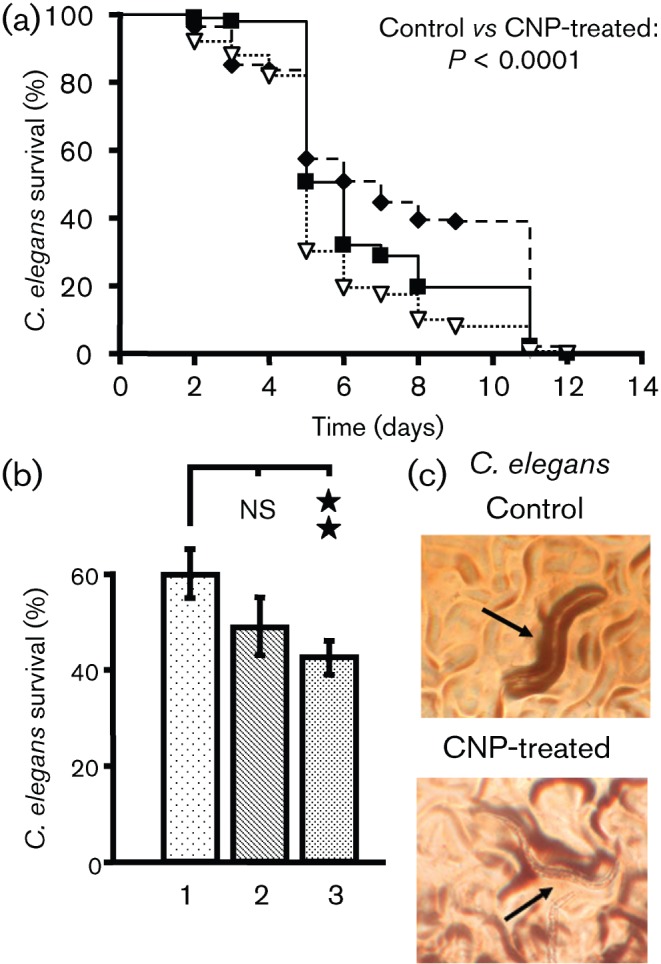
*P. aeruginosa* virulence towards *C. elegans* worms. (a) Slow killing. Kaplan–Meier survival plots of worms fed with *P. aeruginosa* PAO1 control (*n* = 97) (▪, solid line), BNP-treated PAO1 (*n* = 195) (⧫, dashed line) or CNP-treated PAO1 (*n* = 149) (▿, dotted line). Each value is the mean of measurements of nine samples from three independent experiments. Pairwise comparisons (log–rank test) by strain: PAO1 control versus PAO1 CNP-treated, *P*<0.0001; PAO1 control versus PAO1 BNP-treated, *P* = 0.035. (b) Fast-killing. Worm survival after 48 h exposure to *P. aeruginosa* PAO1 control (1) or PAO1 treated with BNP (2) or CNP (3). Each value is the mean±sem of measurements of eight samples from three independent experiments. The non-parametric Mann–Whitney test was used to compare the means within the same set of experiments. Asterisks indicate significant difference (**P<0.01). ns, Not significantly different. (c) Representative pictures of the worm's (indicated by black arrows) state in each condition.

When feeding worms with untreated PAO1 (control), it took 6 days to kill 50 % of *C. elegans* (Fig. 1a). Using PAO1 treated with CNP (1 µM) as food, the worms died more rapidly and only 30 % of worms were alive at day 5 (Fig. 1a). Using the statistical log–rank test we observed that CNP-treated bacteria were more virulent to *C. elegans* than the control bacteria (PAO1 control versus CNP-exposed PAO1; *P* = 0.0002). In contrast, bacteria exposed to BNP (10^−6^ M) appeared less virulent towards *C. elegans* with a median survival at day 7 (Fig. 1a).

*P. aeruginosa* is also able to kill *C. elegans* within a few hours (‘fast killing’) by producing a number of diffusible toxins, including phenazines (Aballay *et al.*, 2003; Mahajan-Miklos *et al.*, 1999; Tan *et al.*, 1999) or HCN (Gallagher & Manoil, 2001). This phenomenon is observed when bacteria are spread in a lawn onto a high-osmolarity medium. When *C. elegans* was dropped on PAO1 control bacteria grown on a high-osmolarity medium, we observed that 60.8±5.2 % of worms were still alive after 48 h (Fig. 1b). The percentage of living worms on CNP-treated bacteria fell to 43.4±3.6 % (Fig. 1b). When *C. elegans* was dropped on BNP-exposed bacteria we observed a slight, but not significant, decrease in the number of worms surviving after 48 h (49.6±5.9 %) (Fig. 1b). Dead worms were stiff and colourless, whereas living worms were dark and curved (Fig. 1c).

### Effect of natriuretic peptides on HCN production by PAO1

In order to identify virulence factors produced by PAO1 after exposure to BNP and CNP that are involved in *C. elegans* killing, we investigated the effect of natriuretic peptides on HCN,) which is the primary diffusible toxic factor that PAO1 produces to kill *C. elegans* (Gallagher & Manoil, 2001).

**Fig. 2.**
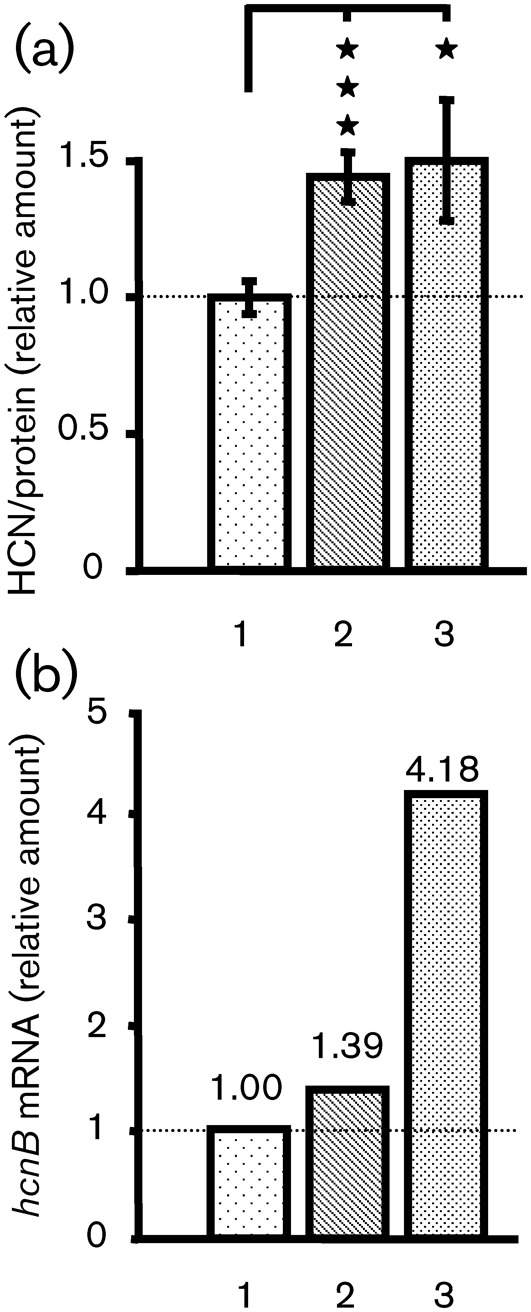
Effect of BNP and CNP on HCN production by *P. aeruginosa* PAO1. (a) Relative amounts of HCN in *P. aeruginosa* PAO1 supernatants, 3 h after bacteria were exposed to physiological water (1), BNP (2) or CNP (3). Data are the mean of five independent experiments. The mean HCN level in the control was 1075±149 µg l^−1^. Asterisks indicate significant difference; ***P<0.001; *P<0.05. (b) *hncB* mRNA levels in treated bacteria relative to those in PAO1 control. RNA was extracted 1 h after exposure of PAO1 to physiological water (1), BNP (2) or CNP (3) and assayed by qRT-PCR. Relative values greater than 1 indicate an increase in gene expression in PAO1-treated bacteria. 16S rRNA was used as an endogenous control to normalize the RNA input and reverse transcription efficiency. qRT-PCRs were performed in triplicate and the sds were lower than 0.15 C_T_. These data are representative of two independent experiments.

The amount of HCN was measured after exposure of bacteria (3 h) to BNP, CNP (both 10 µM) or physiological water (control) during their exponential phase of growth. The results were standardized by dividing the amount of HCN (µg l^−1^) measured in each fraction by the quantity of bacterial proteins from which the fraction was obtained. The treatment of *P. aeruginosa* PAO1 with BNP or CNP significantly increased HCN production, which reached 144.0±8.7 % and 149.7±22.3 % of the control value, respectively (Fig. 2a). In order to determine the origin of the increase in HCN production, we examined the *hcnB* mRNA levels by qRT-PCR 1 h after exposing bacteria to BNP or CNP. The *hcnB* mRNA level rose (4.18-fold) following CNP exposure compared with untreated PAO1, whereas BNP did not significantly affect the level of *hcnB* mRNA (Fig. 2b).

### Effect of natriuretic peptides on production of pyocyanin, PQS and PQS-related molecules by PAO1

Pyocyanin is a virulence factor involved in the lethal activity of *P. aeruginosa* on *C. elegans*. The impact of pyocyanin, however, depends on the *P. aeruginosa* strain. For instance, in strain PA14, pyocyanin production triggers *C. elegans* death (Mahajan-Miklos *et al.*, 1999) whereas in PAO1 there is apparently no correlation between pyocyanin and worm killing (Gallagher & Manoil, 2001). We nevertheless investigated whether pyocyanin could be involved in *C. elegans* killing by PAO1 in our culture conditions and in the pro-virulent effect of CNP.

**Fig. 3.**
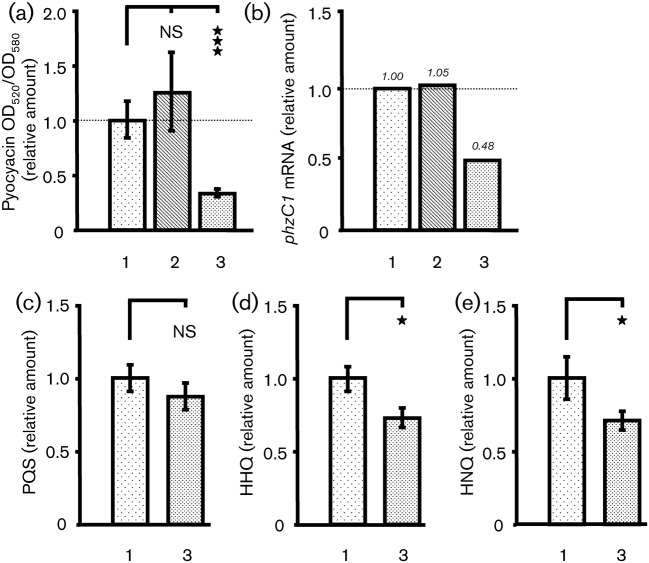
Effect of BNP and CNP on pyocyanin, PQS, HHQ and HNQ production by *P. aeruginosa* PAO1. (a) *P. aeruginosa* PAO1 pyocyanin production in response to natriuretic peptides (BNP and CNP). Data are the mean±sem of seven values obtained from three independent experiments. The mean pyocyanin level in the control was 0.0071±0.0016 OD_520_/OD_580_. Significant difference is indicated by asterisks (***P<0.001); ns, not significantly different. (b) Expression levels of *phzC1* in PAO1-treated bacteria relative to those in the PAO1 control. RNA was extracted 1 h after PAO1 exposure to physiological water or natriuretic peptides (BNP and CNP) and assayed by qRT-PCR. See the legend to Fig. 2 for further information. (c, d) Relative amounts of PQS and HHQ in *P. aeruginosa* PAO1 supernatant 3 h after exposure of PAO1 to physiological water or CNP. Data are the mean±sd of three independent experiments. The mean PQS and HHQ concentration in control conditions were 741×10^3^ Area/OD_580_ and 1410×10^3^ Area/OD_580_, respectively. *P<0.05; ns, not significantly different. (e) Relative amounts of HNQ in *P. aeruginosa* PAO1 supernatant, 3 h after exposure of PAO1 to physiological water or CNP. Data are the means of three independent experiments. The mean HNQ concentration in the control was 1520×10^3^ Area/OD_580_. *P<0.05. 1, Control; 2, BNP-treated PAO1; 3, CNP-treated PAO1.

We first observed that the *phnB* mutant of PAO1 grown in ONB medium killed *C. elegans* with the same kinetics as the wild-type strain (data not shown). Pyocyanin production was measured in culture supernatant (24 h, 37 °C) after exposure of bacteria to BNP or CNP (10^−6^ M). We observed a decrease in pyocyanin production of 65.1±4.3 % for CNP-treated bacteria (Fig. 3a). In contrast, when bacteria were exposed to BNP, no significant variation in pyocyanin was observed (Fig. 3a). The *phzC1* mRNA level was halved after CNP exposure compared with untreated PAO1, whereas BNP did not affect the amount of *phzC1* mRNA (Fig. 3b).

Pre-treatment of *P. aeruginosa* PAO1 with CNP induced a slight decrease in the capacity of the bacteria to produce PQS. Three hours after CNP exposure, PQS production was lowered by 12.3±11.7 % compared with the control value (Fig. 3c). HHQ production by bacteria exposed to CNP was significantly lowered, by 26.7±7.1 %, compared with the control value (Fig. 3d). Pre-treatment of *P. aeruginosa* PAO1 with CNP also induced a significant decrease in the bacterial capacity to produce HNQ. Three hours after exposure to CNP, HNQ production was reduced in the same range as was HHQ (a 29.2±7.0 % decrease compared with the control value) (Fig. 3e).

### Effect of natriuretic peptides on HSL production by PAO1

**Fig. 4.**
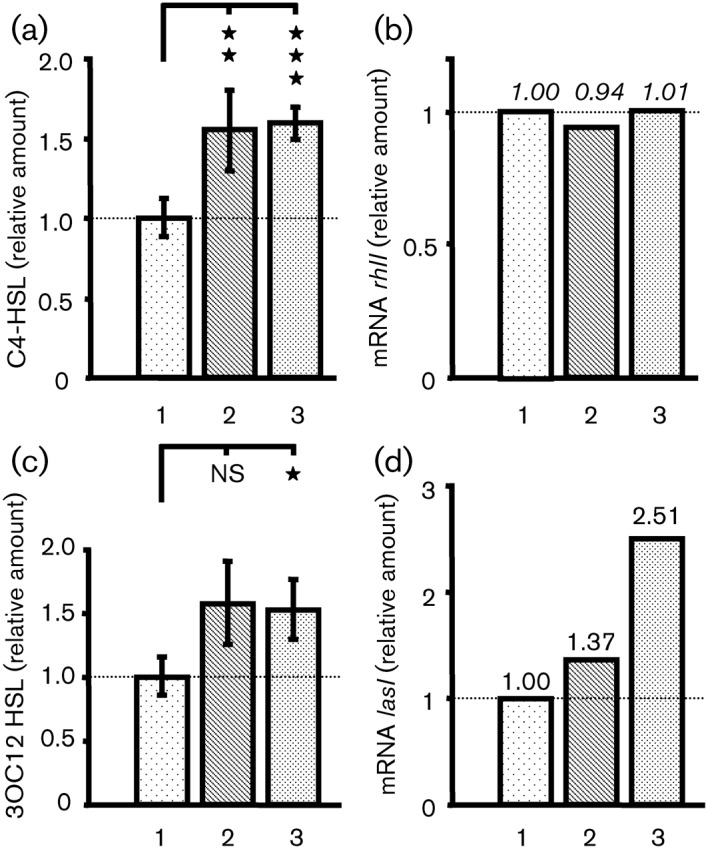
Effect of BNP and CNP on AHL production by *P. aeruginosa* PAO1. (a) and (c) Relative amounts of C4-HSL (a) and 3OC12-HSL (c) in *P. aeruginosa* PAO1 supernatants 3 h after exposure to physiological water (1) or natriuretic peptides [BNP (2) and CNP (3)]. Data are the mean±sem of 11 values obtained from five independent experiments for C4-HSL and of eight values obtained from four independent experiments for 3OC12-HSL. The mean C4-HSL and 3OC12-HSL levels in control conditions were 188.4±25.8 µM and 19.2±2.9 µM, respectively. Significant difference is indicated by asterisks: *P<0.05; **P<0.01; ***P<0.001. (b) and (d) Expression levels of *rhlI* and *lasI* in PAO1-treated bacteria relative to those in the PAO1 control. RNA was extracted 1 h after exposure of PAO1 to physiological water or natriuretic peptides (BNP and CNP) and assayed by qRT-PCR. See the legend to Fig. 2 for further information.

The *P. aeruginosa* PAO1 QS systems LasI/LasR and RhlI/RhlR regulate virulence factor production (Smith & Iglewski, 2003) and are involved in the bacterial killing of *C. elegans* (Darby *et al.*, 1999). The amount of C4-HSL and 3OC12-HSL produced by *P. aeruginosa* was measured after exposure of bacteria to BNP, CNP or physiological water for 3 h during their exponential phase of growth. Pre-treatment of *P. aeruginosa* with either BNP or CNP led to an increase in C4-HSL production compared with untreated bacteria of 156.2±23.7 % and 160.6±10.6 %, respectively (Fig. 4a). We observed that the increase in C4-HSL production was maintained 5 h after exposure to BNP and CNP, and the levels of C4-HSL returned to the control values 9 h after the onset of the experiment (data not shown). We examined the *rhlI* mRNA levels by qRT-PCR. RNA was extracted from bacteria harvested after 1 h of exposure to BNP, CNP or physiological water. We observed that the *rhlI* mRNA levels were not modified by exposure of PAO1 to BNP or CNP compared with untreated bacteria (Fig. 4b).

Pre-treatment of *P. aeruginosa* with BNP and CNP for 3 h also induced an increase in the capacity of the bacteria to produce 3OC12-HSL. Bacteria exposed to BNP and CNP showed an increase in 3OC12-HSL production which reached 156.0±33.7 % and 150.1±25.6 % of the control value (Fig. 4c). This increase in 3OC12-HSL production was maintained up to 5 h after exposure of PAO1 to BNP and CNP, and returned to the basal level within 9 h (data not shown). We examined the *lasI* mRNA levels by qRT-PCR. While the *lasI* mRNA level slightly increased after BNP exposure (×1.37), PAO1 exposure to CNP induced a significantly higher rise in *lasI* mRNA levels (×2.51) compared with untreated PAO1 (Fig. 4d).

**Fig. 5.**
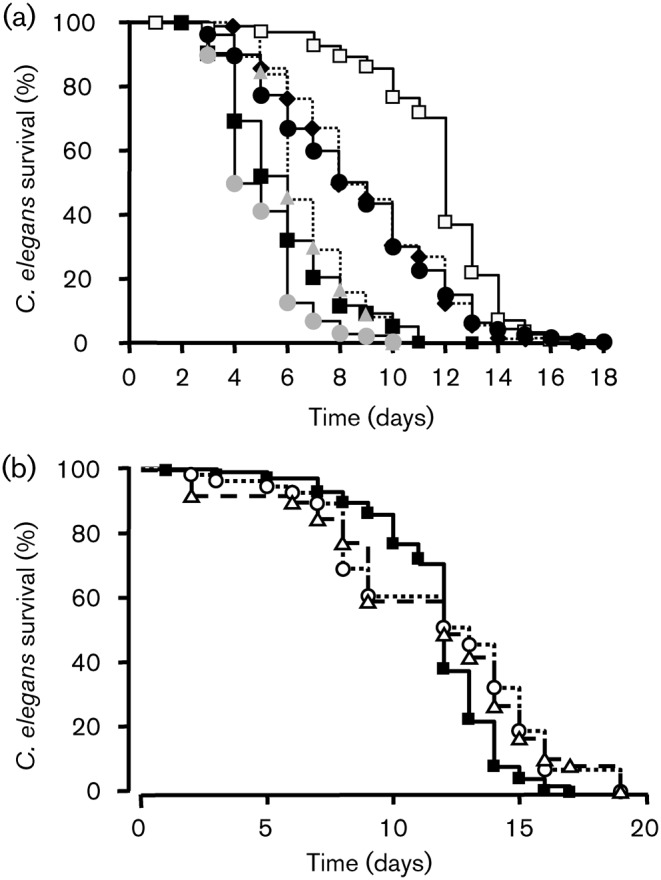
Role of the LasI/LasR and RhlI/RhlR QS systems and HSL on the capacity of PAO1 to kill *C. elegans*. (a) Slow killing. Kaplan–Meier survival plots of worms fed with *P. aeruginosa* MPAO1 control (*n* = 401) (▪), *lasR* mutant (*n* = 243) (•), *rhlR* mutant (*n* = 262) (⧫), *lasI* mutant (*n* = 105) (•), *rhlI* mutant (*n* = 90) (▴) or *E. coli* OP50 (*n* = 212) (□). Each value is the mean of measurements from nine samples from three independent experiments. (b) Slow killing. Kaplan–Meier survival plots of worms fed with *E. coli* OP50 control (*n* = 212) (▪), *E. coli* OP50 with 0.5 mg C4-HSL l^−1^ (*n* = 82) (○) or *E. coli* OP50 with 0.5 mg 3OC12-HSL l^−1^ (*n* = 59) (▵). Each value reported for the assay is the mean of measurements from three samples.

Using mutants, we evaluated the involvement of LasI/LasR and RhlI/RhlR systems on the lethal activity of PAO1 on *C. elegans* after growth in ONB medium. We first observed that *lasI* and *rhlI* mutants possessed a capacity to kill *C. elegans* in the same range as the wild-type (Fig. 5a). In contrast, *lasR* and *rhlR* mutants appeared less virulent than the wild-type strain (Fig. 5a).

In order to evaluate a direct toxic effect of HSLs on *C. elegans*, we exposed worms to *E. coli* OP50 cultivated on NGM plates supplemented with C4-HSL or 3OC12-HSL (0.5 mg l^−1^). We observed that neither C4-HSL nor 3OC12-HSL had a significant effect on *C. elegans* survival kinetics (Fig. 5b).

### Effect of natriuretic peptides on ETA production by PAO1

*P. aeruginosa* secretes numerous extracellular products, including ETA, elastase and protease, that are important for virulence and that are regulated by QS molecules (de Kievit & Iglewski, 2000). The treatment of *P. aeruginosa* PAO1 with BNP or CNP for 3 h did not modify elastase and protease (azocasein hydrolytic) activities of the bacteria (data not shown).

**Fig. 6.**
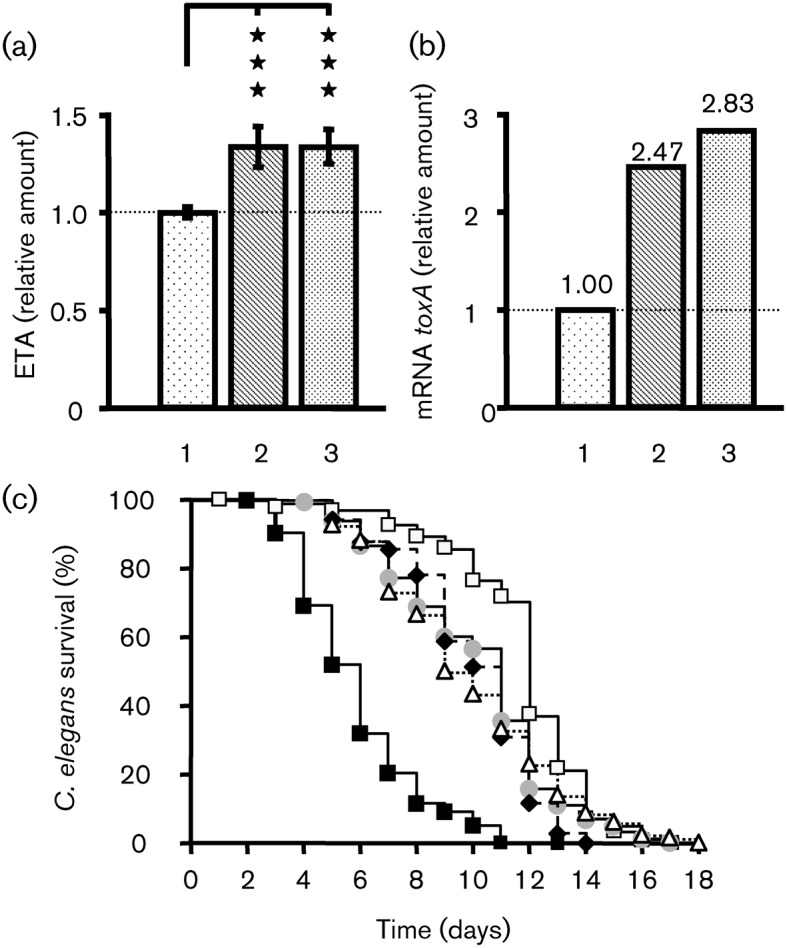
Effect of BNP and CNP on ETA production by *P. aeruginosa* PAO1. (a) Relative amounts of ETA in *P. aeruginosa* PAO1 supernatants 3 h after exposure of bacteria to physiological water or natriuretic peptides (BNP or CNP). Data are the mean±sem of five independent experiments. The mean ETA level in the control was 33.6±4.0 µg µl^−1^ OD_580_^−1^. ***P<0.001. (b) Expression levels of *toxA* genes in PAO1-treated bacteria relative to those in the PAO1 control. RNA was extracted 1 h after PAO1 exposure to physiological water or natriuretic peptides (BNP or CNP) and assayed by qRT-PCR. See the legend to Fig. 2 for further information. These data are representative of two independent experiments. 1, Control; 2, BNP-treated PAO1; 3, CNP-treated PAO1. (c) Slow killing. Kaplan–Meier survival plots of worms fed with *P. aeruginosa* MPAO1 control (*n* = 401) (▪), *toxR* mutant control (*n* = 149) (•), BNP-treated *toxR* mutant (*n* = 68) (⧫), CNP-treated *toxR* mutant (*n* = 78) (▵) or *E. coli* OP50 (*n* = 212) (□). Each value is the mean of measurements from three samples from two independent experiments. Pairwise comparisons (log–rank test) of wild-type versus *toxR* mutant control, *P*<0.0001.

The amount of ETA produced by *P. aeruginosa* PAO1 was determined by ELISA (Coligan *et al.*, 2001). The results were standardized by dividing the amount of ETA (pg ml^−1^) measured in each fraction by the OD_580_ of the culture medium from which the fraction was obtained. The treatment of *P. aeruginosa* PAO1 with BNP or CNP for 3 h significantly increased ETA production, which reached 127.1±8.4 % and 129.7±8.1 % of the control values, respectively (Fig. 6a).

In order to determine the origin of the increase in ETA production, we examined the *toxA* mRNA levels by qRT-PCR. The *toxA* mRNA level was increased by exposure to either BNP or CNP compared with untreated PAO1 (×2.47 and ×2.83, respectively) (Fig. 6b). ToxR (RegA) is a regulator of ETA production in PAO1. We observed that a *toxR* (*regA*) mutant lost all lethal activity in the *C. elegans* model and that both BNP and CNP are unable to increase *toxR* mutant virulence (Fig. 6c).

### Implication of Vfr in PAO1 response to natriuretic peptides

The protein Vfr is a global regulator of PAO1 virulence and is activated by cAMP. Since it has been shown that CNP induces a twofold increase of cAMP into PAO1 (Veron *et al.*, 2007), we investigated the role of Vfr in the CNP pro-virulence effect in the *C. elegans* model.

**Fig. 7.**
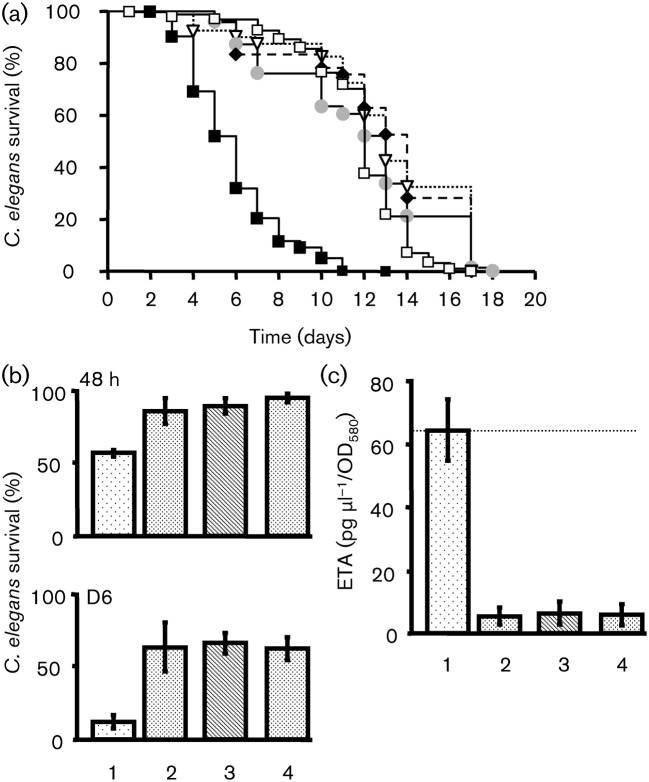
Role of Vfr in the effect of natriuretic peptides on PAO1. (a) Slow killing. Kaplan–Meier survival plots of worms fed with *P. aeruginosa* MPAO1 control (*n* = 401) (▪), *vfr* mutant control (*n* = 73) (•), BNP-treated *vfr* mutant (*n* = 78) (⧫), CNP-treated *vfr* mutant (*n* = 40) (▿) or *E. coli* OP50 (*n* = 212) (□). Each value is the mean of at least three samples. Pairwise comparisons (log–rank test) of wild-type versus *vfr* mutant control, *P*<0.0001. (b) Fast-killing. Worm survival after 48 h or 6 days, (D6) exposure to *P. aeruginosa* MPAO1 control (*n* = 291; 1), *vfr* mutant control (*n* = 94; 2), BNP-treated *vfr* mutant (*n* = 106; 3) or CNP-treated *vfr* mutant (*n* = 112; 4). Each value reported is the mean±sem of six samples from two independent experiments. (c) ETA in supernatants from *P. aeruginosa* MPAO1, *vfr* mutant control, BNP-treated *vfr* mutant or CNP-treated *vfr* mutant. Bars are numbered as in (b). Data are the means±sem of four independent experiments. The mean ETA level in the control was 63.6±9.7 µg µl^−1^ OD_580_^−1^.

**Fig. 8.**
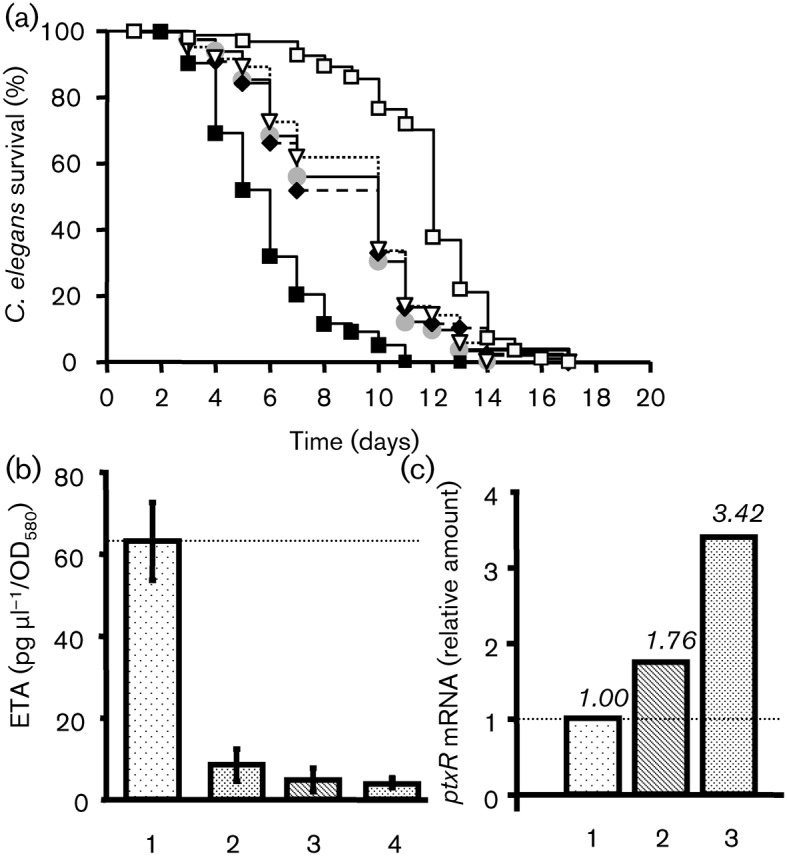
Role of PtxR in the effect of natriuretic peptides on PAO1. (a) Slow killing. Kaplan–Meier survival plots of worms fed with *P. aeruginosa* MPAO1 control (*n* = 401) (▪), *ptxR* mutant control (*n* = 82) (•), BNP-treated *ptxR* mutant (*n* = 77) (⧫), CNP-treated *ptxR* mutant (*n* = 84) (▿) or *E. coli* OP50 (*n* = 212) (□). Each value is the mean from three samples. Pairwise comparisons (log–rank test) of wild-type versus *ptxR* mutant control, *P*<0.0001. (b) Amount of ETA in supernatant from *P. aeruginosa* MPAO1 (1), *ptxR* mutant control (2), BNP-treated *ptxR* mutant (3) or CNP-treated *ptxR* mutant (4). The mean ETA level in the control was 63.6±9.7 µg µl^−1^ OD_580_^−1^. Data are the mean±sem of three independent experiments. (c) Expression levels of *ptxR* in treated bacteria relative to those in PAO1 control. RNA was extracted 1 h after exposure of PAO1 physiological water (1) or natriuretic peptides [BNP (2) and CNP (3)] and assayed by qRT-PCR. These data are representative of two independent experiments. See the legend to Fig. 2 for further information.

We observed that the *vfr* mutant dramatically lost its ability to kill *C. elegans* in both slow-killing (Fig. 7a) and fast-killing (Fig. 7b) tests. When the *vfr* mutant was exposed to BNP or CNP, we observed no modification in the virulence activity of this strain (Figs 7a and 8a). In addition, we noted that the *vfr* mutant produced a small amount of ETA, the levels of which remained unchanged even after exposure to BNP or CNP (Fig. 7c).

### Role of PtxR in the effect of natriuretic peptides on PAO1

PtxR regulates ETA production in PAO1 (Hamood *et al.*, 1996) and is under the control of Vfr (Ferrell *et al.*, 2008). We decided to evaluate the role of the PtxR regulator on the effect of CNP on PAO1.

We observed that the *ptxR* mutant partially lost its virulence towards *C. elegans* in the slow-killing model (Fig. 8a). In contrast, the virulence of this mutant was retained in a fast-killing test (data not shown). When the *ptxR* mutant was exposed to BNP or CNP, we observed no modification of the virulence pattern of this strain (Fig. 8a). In addition, we remarked that, as in the *vfr* mutant, the level of ETA produced by the *ptxR* mutant was low and not affected by BNP and CNP (Fig. 8b). We examined *ptxR* mRNA levels by qRT-PCR. The level of *ptxR* mRNA increased slightly after exposure to BNP (×1.76), whereas exposure to CNP had a more pronounced effect (the *ptxR* mRNA level was 3.42 times greater than that of untreated PAO1) (Fig. 8c).

### Role of *algC* in the effect of natriuretic peptides on PAO1

**Fig. 9.**
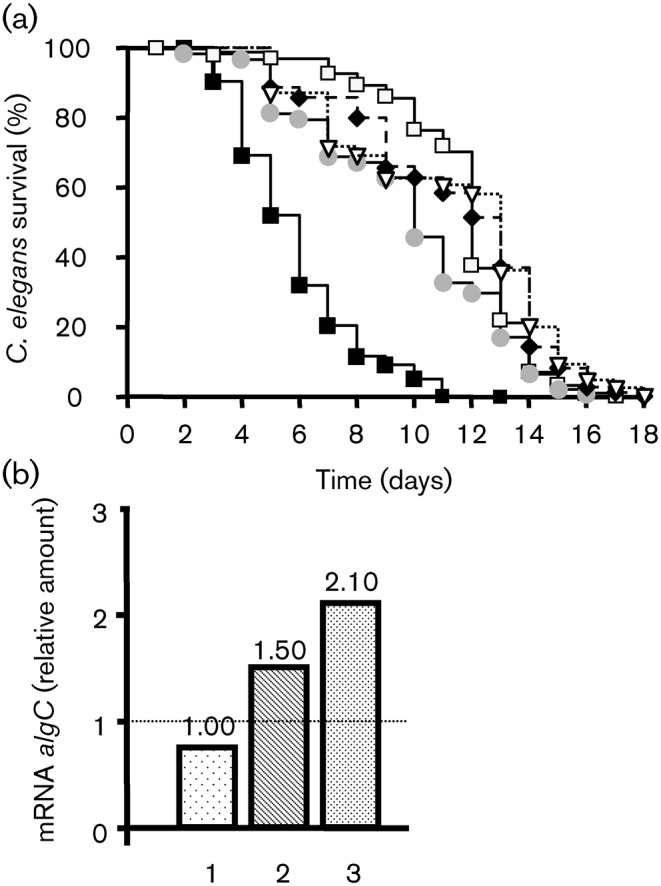
Role of AlgC in the effect of natriuretic peptides on PAO1. (a) Slow killing. Kaplan–Meier survival plots of worms fed with *P. aeruginosa* MPAO1 control (*n* = 401) (▪), *algC* mutant control (*n* = 182) (•), BNP-treated *algC* mutant (*n* = 102) (⧫), CNP-treated *algC* mutant (*n* = 67) (▿) or *E. coli* OP50 (*n* = 212) (□). Each value is the mean±sem from three samples. Pairwise comparisons (log–rank test) of wild-type versus *algC* mutant control, *P*<0.0001. (b) Expression levels of *algC* in treated bacteria relative to those in the PAO1 control. RNA was extracted 1 h after exposure of PAO1 to physiological water (1) or natriuretic peptides [BNP (2) and CNP (3)] and assayed by qRT-PCR. These data are representative of two independent experiments. See the legend to Fig. 2 for further information.

It has been shown that cAMP, after binding to Vfr, modulates the structure of LPS in PAO1 (Veron *et al.*, 2007) and that *algC* is required for the synthesis of a complete LPS core, which is involved in the lethal effect of bacteria on *C. elegans* (Gallagher & Manoil, 2001). We therefore investigated the role of *algC* in the pro-virulence effect of CNP on PAO1. We observed that the *algC* mutant grown in ONB medium dramatically lost its ability to kill *C. elegans* in the slow-killing conditions (Fig. 9a). When the *algC* mutant was exposed to BNP or CNP we observed no modification in the virulence activity of this strain (Fig. 9a). In addition, the level of *algC* mRNA measured by qRT-PCR was not significantly modified after BNP exposure (×1.50) whereas CNP exposure doubled the *algC* mRNA level compared with untreated PAO1 (Fig. 9b).

## DISCUSSION

Originally, we described that natriuretic peptides, in particular CNP, enhance *P. aeruginosa* PAO1 cytotoxicity through the activation of Vfr, a global regulator of bacterial virulence (Veron *et al.*, 2007). Since the pro-cytotoxic effect of natriuretic peptides was only observed *in vitro*, we decided to investigate further the mechanism of action of these molecules by evaluating their impact on bacterial virulence using the more complex pluricellular model *C. elegans*. In the present study, we provide evidence that CNP enhances the global virulence of PAO1 to *C. elegans*, by increasing the secretion of HCN and ETA through stimulation of the regulatory proteins Vfr and PtxR and activation of QS.

Our experiments indicate that CNP is involved in the regulation of the virulence of *P. aeruginosa* to *C. elegans*, both through the infection-like process (slow killing) and the production of diffusible toxins (fast killing). The lethal effect of *Pseudomonas* on *C. elegans* is multifactorial but HCN appears to be the primary diffusible toxic factor killing *C. elegans* (Gallagher & Manoil, 2001). The lethal effect of *Pseudomonas* on *C. elegans* could also be mediated by the blue phenazine pigment pyocyanin, which is known to be essential in the *P. aeruginosa* PA14 fast-killing test (Mahajan-Miklos *et al.*, 1999). Pyocyanin is the major phenazine produced by *P. aeruginosa* and it acts as a virulence factor (Lau *et al.*, 2004) that is directly toxic to isolated eukaryotic cells (Denning *et al.*, 1998), prokaryotic cells (Hassan & Fridovich, 1980) and complex organisms such as *C. elegans* (Mahajan-Miklos *et al.*, 1999). We observed that pyocyanin produced by PAO1 in ONB medium (this study) is not involved in *C. elegans* killing, confirming that PAO1 does not act on *C. elegans* through this diffusible factor (Gallagher *et al.*, 2002). However, we noted that although pyocyanin is not involved in the PAO1 killing activity, CNP modulates pyocyanin production. This result is consistent with our hypothesis that CNP acts on *Pseudomonas* through different mechanisms and toxic molecules.

Bacteria must integrate extra- and intracellular information to respond appropriately to environmental changes (Miller *et al.*, 1989). The pathways by which pathogenic bacteria are activated by host signals appear to be, like receptor–ligand interactions, specific to bacteria and to the host signal involved (for a review see Lesouhaitier *et al.*, 2009). Concerning *P. aeruginosa*, the sensitivity to eukaryotic signal molecules comes with the regulation of bacterial QS molecule production (Wu *et al.*, 2005; Zaborina *et al.*, 2007). Since QS-dependent exoproducts such as pyocyanin production are under the influence of PQS (Déziel *et al.*, 2004; Gallagher *et al.*, 2002), we evaluated the impact of CNP on the production of PQS and related molecules. By analysing the PQS production profile as a function of growth, it has been suggested that PQS is not maximally produced until the late stationary phase and that very little PQS is present when the bacterial cells enter the stationary phase (McKnight *et al.*, 2000). However, although PQS levels are indeed maximal in late stationary phase, the molecule is already detectable in the exponential phase of growth, and it was demonstrated that PQS, apart from being important in the late stationary phase, may also have a function much earlier in the growth phase (Diggle *et al.*, 2003; Guina *et al.*, 2003). Then, we measured the impact of CNP on the capacity of *P. aeruginosa* PAO1 to produce PQS and its precursor HHQ at the end of the exponential growth phase. We observed that CNP slightly reduces PQS production, apparently as a consequence of a significant decrease in its precursor HHQ synthesis. This hypothesis is supported by the observation that a reduction in HNQ, which is derived from HHQ, as is PQS. Nevertheless, these results indicate that the effect of CNP on PAO1 virulence is not mainly mediated by the PQS system.

The production of PQS in *P. aeruginosa* is finely regulated and interconnected with cell-to-cell signals AHLs (Wade *et al.*, 2005). Among AHLs, the two chemically distinct molecules (C4-HSL, 3OC12-HSL), which act in concert with PQS, control the expression of many genes that are eventually involved in the global virulence of the bacterium (Diggle *et al.*, 2007; Xiao *et al.*, 2006). We observed that the pro-virulent effects of natriuretic peptides on *P. aeruginosa* PAO1 could be supported by modifications of C4-HSL and 3OC12-HSL production. Both BNP and CNP significantly enhance the bacterial production of C4-HSL and 3OC12-HSL. These effects were observed during the end of the exponential phase and 3 h after bacterial exposure to BNP or CNP, and appeared transient, since the levels of C4-HSL and 3OC12-HSL returned to the control values 9 h later. These results are consistent with previous studies showing that the cytotoxic effect of PAO1 is enhanced by BNP and CNP (Veron *et al.*, 2007) and we suggest that the increase in AHL supports a part of the pro-virulent action of natriuretic peptides on *P. aeruginosa* PAO1. It has been proposed that 3OC12-HSL, in addition to its informative activity, can act by itself as a virulence factor able to induce inflammation *in vivo* (Smith *et al.*, 2002), to accelerate apoptosis in macrophages and neutrophils (Tateda *et al.*, 2003), and to interact directly with immune cell membranes (Davis *et al.*, 2010). However, we verified that both 3OC12-HSL and C4-HSL are unable to kill *C. elegans* directly, indicating that HSL are not or are only slightly toxic on a complex organism such as *C. elegans*. Analysis of the time-course synthesis of HSL in PAO1 showed that 3OC12-HSL is first synthesized under *lasI* activation and that C4-HSL is subsequently produced under *rhlI* activation (Pesci *et al.*, 1997). In our study, we observed that 1 h after PAO1 exposure to CNP, the amount of *lasI* mRNA was enhanced by 2.5-fold whereas the amount of *rhlI* mRNA remained unchanged. The fact that both C4-HSL and 3OC12-HSL are overproduced 3 h after PAO1 exposure to CNP is in agreement with the 3OC12-HSL-dependent mechanism of C4-HSL synthesis and suggests that CNP activates bacterial internal regulators that act through the same cascade of events. Both LasI/R and RhlI/R QS systems of *P. aeruginosa* are involved in *C. elegans* killing (Steindler *et al.*, 2009). We observed that only *lasR* and *rhlR* mutants lose their virulence, whereas the virulence of *lasI* and *rhlI* mutants is preserved, which shows a clear dissociation between the functions of *lasR* and *rhlR* receptor and their classical ligands, as previously suggested (Darby *et al.*, 1999; Steindler *et al.*, 2009). Moreover, these results reinforce the recent hypothesis proposing that LasR can switch into a functional conformation in the absence of an AHL ligand (Sappington *et al.*, 2011).

Among *P. aeruginosa* secreted virulence factors, ETA is the most toxic, and this toxin appears indispensable to the infectious success of the bacterium (Fogle *et al.*, 2002; Hamood *et al.*, 1996; Storey *et al.*, 1998). The production of ETA by *P. aeruginosa* depends on environmental conditions such as iron concentration (Hamood *et al.*, 2004; Liu, 1973), but it was also reported to be regulated by a neurotransmitter such as norepinephrine (Li *et al.*, 2009). We investigated a possible action of natriuretic peptides on ETA production by PAO1. We observed that both BNP and CNP increase the ETA production by PAO1. This effect appears to be correlated with an increase in the transcription of the *toxA* gene, suggesting that ETA synthesis is genomically regulated by natriuretic peptides. It has been shown that, in *P. aeruginosa* PA14, ETA is involved in *C. elegans* fast and/or slow killing (Tan *et al.*, 1999). Using a *toxR* mutant, we confirmed that ETA from *P. aeruginosa* PAO1 is at least partially involved in the effect of CNP on virulence in this strain, indicating that, in PA14 as in PAO1, ETA supports the killing of *C. elegans*. In contrast, elastase and protease appear to have no role in the pro-virulence effect of CNP on PAO1 in the *C. elegans* model.

Whereas only CNP actually enhances PAO1 virulence, we noticed that BNP is also able to induce ETA and HSL production by the bacteria. Three subtypes of natriuretic peptide receptors (NPRs) are expressed on eukaryotic cell membranes. Of these receptors, two (NPR-B and NPR-C) are preferentially activated by CNP rather than BNP (Potter *et al.*, 2006). Our results suggest that the PAO1 natriuretic peptide sensor could be related to one of these two NPR subtypes. Nevertheless, these NPRs can also be activated, with a lower efficiency, by BNP, and this difference could explain how, in our study, the effect of BNP on PAO1 toxin production is limited compared with CNP. Furthermore, we cannot rule out the presence of different subtypes of *Pseudomonas* natriuretic peptide sensors, as observed in eukaryotic cells (Anand-Srivastava *et al.*, 1996; Potter & Hunter, 2001). Indeed, we observed previously that the sensitivity to natriuretic peptides diverges between *Pseudomonas* species, for instance between *P. fluorescens* and *P. aeruginosa* (Veron *et al.*, 2008). Consistent with this notion of high stereospecific recognition of eukaryotic messengers in bacteria, it has been shown that *Helicobacter pylori* can recognize only one of the subtypes of the somatostatin receptor agonists (Yamashita *et al.*, 1998).

**Fig. 10.**
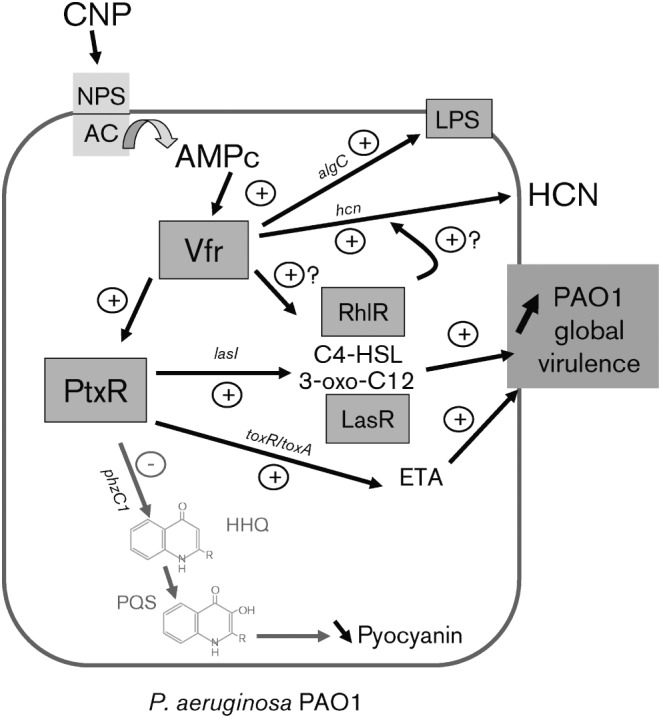
Schematic model representing the mechanism of action of natriuretic peptides on *P. aeruginosa* PAO1. Activation of the membrane natriuretic peptides sensor (NPS) by CNP induces a rise in intra-bacterial cAMP concentration through adenylate cyclase (AC) activation (Veron *et al.*, 2007). Then, the global regulator Vfr, activated by cAMP, regulates directly or indirectly (after PtxR activation) the expression of several virulence factors such as ETA, HCN and pyocyanin through the modulation of QS signalling molecules. Finally, CNP binding on *P. aeruginosa* PAO1 increases global bacteria virulence.

In order to go further into the mechanism of action of natriuretic peptides in *P. aeruginosa* we decided to investigate the transcriptional regulator(s) potentially involved in the effect of CNP. In *P. aeruginosa*, numerous proteins such as Vfr, GacA, MvaT, QscR, PtxR, RsaL, VqsM and VqsR regulate (positively or negatively) the expression of QS genes (Albus *et al.*, 1997; Chugani *et al.*, 2001; Diggle *et al.*, 2002; Dong *et al.*, 2005; Juhas *et al.*, 2004; Reimmann *et al.*, 1997; Schuster & Greenberg, 2006). ETA synthesis itself is controlled both by a cascade of positive regulators, including PvdS and RegA (ToxR) protein (Hamood *et al.*, 2004; Ochsner *et al.*, 1996) or the PtxR regulator (Hamood *et al.*, 1996), and also by direct binding of the PAO1 global virulence regulator Vfr on the *toxA* promoter region (Davinic *et al.*, 2009). We have demonstrated previously that the Vfr protein is involved in the effect of natriuretic peptides on *P. aeruginosa* PAO1 virulence (Veron *et al.*, 2007). We observed here that Vfr is not only essential for expression of the lethal effect of PAO1 on *C. elegans* in both fast and slow killing conditions but also involved in the pro-virulent effect of BNP and CNP. Since the PtxR regulator, which regulates ETA production in PAO1 (Hamood *et al.*, 1996), is under the control of Vfr (Ferrell *et al.*, 2008), and ETA synthesis is promoted by CNP (and BNP) we naturally examined the role of PtxR in CNP action. We observed that CNP induced a 3.5-fold increase of *ptxR* mRNA synthesis 1 h after its contact with PAO1, suggesting that PtxR is involved in the effect of CNP on PAO1. Moreover, the observation that PtxR negatively regulates the expression of pyocyanin while it positively regulates the production of 3OC12-HSL through *lasI* (Carty *et al.*, 2006) strongly reinforces our hypothesis that PtxR is a member of the transduction cascade triggered by CNP in PAO1 (Fig. 10). However, PtxR appears to regulate only a part of the effect of CNP, since a *ptxR* mutation does not lead to a total suppression of bacterial virulence as observed with the *vfr* mutant, and whereas PtxR is necessary for the expression of the slow-killing activity of PAO1, this protein is not required to kill *C. elegans* in the fast-killing test (data not shown). We know that Vfr is involved in LPS structural rearrangements induced by CNP (Veron *et al.*, 2007) and an *algC* mutant is found to be avirulent, an effect which has been specifically ascribed to LPS biosynthesis modifications (Goldberg *et al.*, 1995). In the present study, we observed that not only is an *algC* mutant less virulent towards *C. elegans* compared with the wild-type but also CNP pro-virulent activity could be assigned to LPS through *algC* since CNP enhanced the *algC* mRNA level in PAO1. It is interesting to note that although the NF-κB signalling pathway is not functional in the immune system of *C. elegans* (Wang *et al.*, 2006) and although this worm possesses a single Toll-like receptor homologue (Pujol *et al.*, 2001), this worm could be employed as a model to study pathogenesis supported by LPS.

Iron represses ETA production at the transcriptional level (Lory, 1986). Conversely, PvdS and, to a lesser extent, PtxR enhances the expression of both *toxA* and the genes necessary for production of the siderophore pyoverdine (Hamood *et al.*, 1996, 2004). We cannot therefore rule out that CNP interferes with iron uptake and in return enhances ETA production through the stimulation of PtxR. However, the link between CNP and iron starvation remains speculative, and we did not observe any effect of CNP on pyoverdine synthesis (data not shown).

The present data allowed a global diagram of the mechanism of action of CNP on PAO1 to be proposed, from the binding on a hypothetical membrane sensor to the production of virulence factors (Fig. 10). We observed that CNP triggers the synthesis of mRNA coding numerous proteins. This increase peaked around 1 h after the initial contact with CNP. Since we did not observe mRNA synthesis modification 10 min after CNP administration (data not shown) it appears that between 10 min and 1 h is necessary for transmission of the CNP signal from the membrane to gene expression. As expected for a signal, this phenomenon is transient since we observed that 3 h after CNP exposure, mRNA amounts were still higher, but came closer to the control values (data not shown). This increase in mRNA synthesis was related not to a concomitant enhancement of protein production, but to a maximum level of all measured virulence factors that was observed 3 h after CNP exposure. This relatively long delay is probably explained by the necessity to accumulate factors in bacterial medium at a level that was sufficient for experimental detection. It is interesting to note that the effect of CNP on each individual virulence factor (HCN, ETA, QS molecules) is rather limited, but the global activity of CNP on PAO1 virulence is probably due to additive or even synergistic effects between the different toxins.

Taken together, the present data reveal that in PAO1, the pro-virulent effect of the natriuretic peptide CNP is supported by several factors and involves the relay of different regulatory proteins acting through the stimulation of the HSL systems (Fig. 10). *P. aeruginosa* infections associated with cystic fibrosis are resistant to current therapies, and molecules inhibiting toxin production or signals controlling their production should be able to reduce infection and aid in the clearance of this pathogen. Our study suggests that chemicals able to block or reverse the effect of natriuretic peptides on bacteria may serve as a basis for the development of compounds inhibiting the global virulence of *P. aeruginosa*. These data, added to those of Zaborina *et al.* (2007) on dynorphin or Hegde *et al.* (2009) on norepinephrine, show that QS is involved in the bacterial response to eukaryotic communication molecules and suggest that inter-kingdom signal exchanges are certainly essential in the pathogenic activity of bacteria.
